# Phyllosphere synthetic microbial communities: a new frontier in plant protection

**DOI:** 10.1186/s12870-025-06935-7

**Published:** 2025-07-23

**Authors:** Easton Sarver, Kevin J. González-Morelo, Katie G. Christensen, Hanna M. Lefevers, Kendall R. Corbin

**Affiliations:** Department of Horticulture, Martin-Gatton College of Agriculture, Food and Environment, University of Kentucky, Lexington, KY USA

**Keywords:** Phyllosphere, Phyllosphere-modulating SynCom, Plant protection, Plant health, Biocontrol, Biostimulant, Synthetic microbial community

## Abstract

**Background:**

The phyllosphere, which includes the surfaces of plant leaves and stems, is one of the largest and most diverse microbial habitats on Earth, yet it remains understudied in plant-microbe interaction research. Recent studies have highlighted the significant role of phyllosphere epiphytic bacteria in enhancing plant health. These microorganisms help improve nutrient uptake, defend against pathogens, and increase resilience to environmental stressors.

**Main body:**

In recent years, phyllosphere-associated microorganisms have been assembled into synthetic microbial communities (SynComs) to replicate or augment natural microbial populations. This review examines the emerging field of phyllosphere-modulating synthetic communities (PMS) and their potential to enhance plant fitness and protection. We explore the latest advancements in the design of SynComs, with a focus on their agricultural applications. Despite promising results, a consensus is lacking on best practices for standardizing the development and application of PMS, with the complexity of PMS reported in the literature ranging from a few species to as many as 48 core phyla, including Proteobacteria, Firmicutes, and Actinobacteria.

**Conclusion:**

While PMS present a promising alternative to conventional plant protection methods, their full potential remains underexplored. Continued efforts to standardize and refine phyllosphere-modulating SynComs are essential to establishing them as reliable biological tools for improving plant health.

## Background

Microbial communities, collectively referred to as the microbiota, are groups of microorganisms that co-exist and inhabit a wide range of environments, including plants [1, 2]. These communities play a vital role in promoting plant health, as they engage in complex interactions with their host. For example, plant-associated microorganisms produce hormones, suppress pathogens, and facilitate nutrient acquisition, all of which enhance plant resilience and productivity across diverse environmental conditions [3–5]. Recent advances in microbiome research and sequencing technologies have deepened our understanding of the complexity and importance of these communities. These insights are now paving the way for innovative strategies to harness plant-associated microbes for improved plant health.

One promising area of innovation is the modulation of plant-associated microbiotas using synthetic microbial communities (SynComs). SynComs (also referred to as engineered microbial consortia, microbial assemblages, mock communities, or synthetic microbiomes) are custom-designed groups of microorganisms that are intentionally assembled to mimic or enhance natural microbial communities [6]. SynComs may be designed to represent the core microbiome of the host plant or customized with satellite microbes not native to the environment of interest [7]. These communities are tailored to optimize specific plant functions, offering a novel approach to modulating the plant microbiota for improved crop protection.

Plants, much like humans, host a diverse array of microbial communities, each adapted to specific micro-environments within the plant. These microbial populations colonize distinct ecological niches across various plant compartments, including the rhizosphere (soil), endosphere (roots), and phyllosphere (leaf surfaces) [5, 8].While research efforts have traditionally focused on microbial interactions within the rhizosphere and their applications in soil management [9, 10], there is emerging evidence that phyllosphere-associated microorganisms are important for plant health [11, 12]. The phyllosphere, which encompasses the above-ground surfaces of plants, is one of the largest terrestrial ecosystems, covering an estimated surface area of 1 billion km² globally [13]. Of this above ground biomass, the resource-limited environment of plant leaves can support bacterial populations of up to 10^7^ colony-forming units (CFU) per gram of leaf [14–16]. These microbial communities contribute to plant health by suppressing pathogens, modulating host immunity, and assisting in nutrient cycling [17]. Disturbances in the phyllosphere microbiota, whether due to pathogen invasion, environmental stress, or agricultural practices, can lead to imbalances (dysbiosis) that negatively impact plant health and broader ecological functions, including atmospheric carbon dioxide and oxygen cycles [17, 18].

**Fig. 1:**
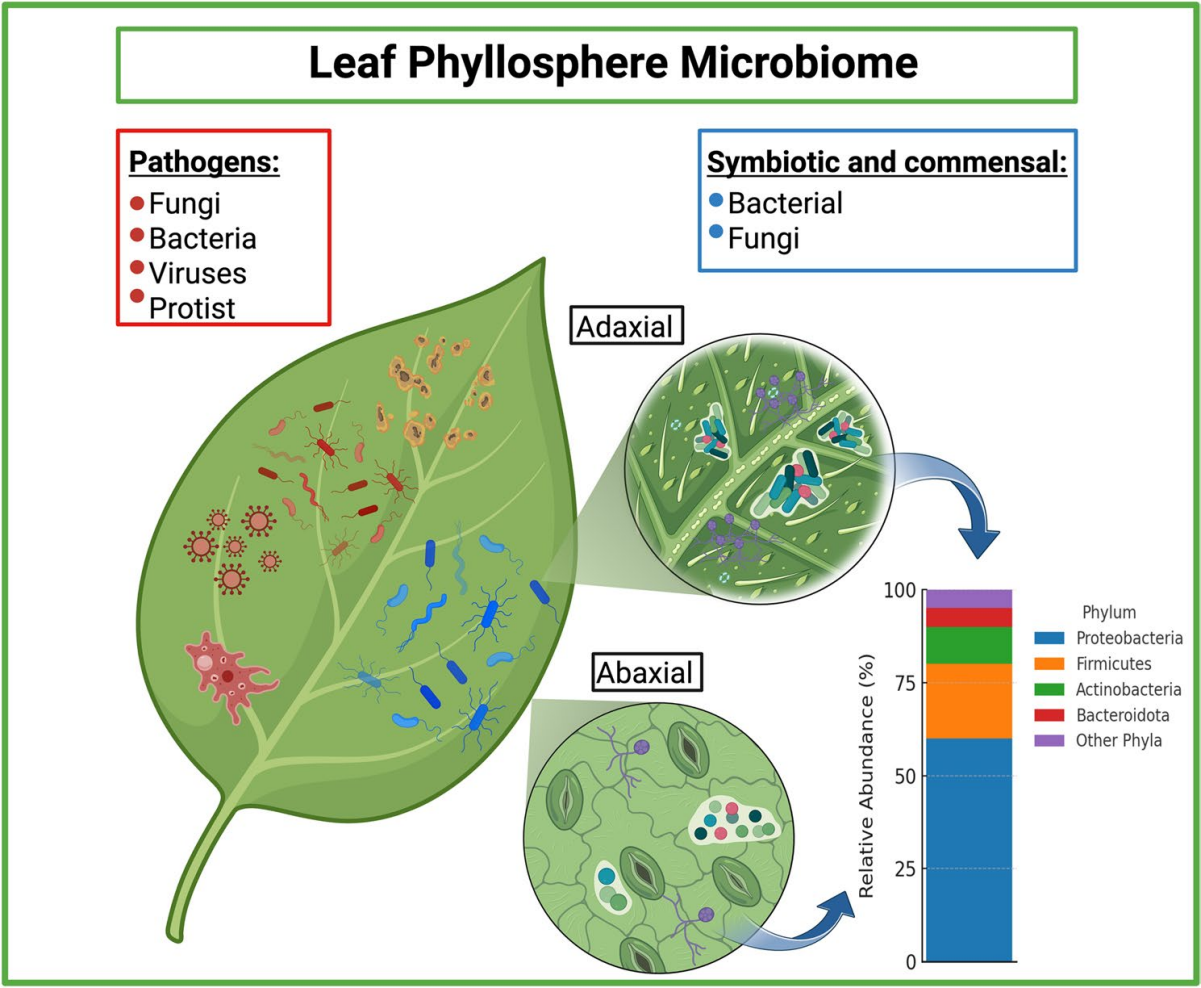
The plant leaf phyllosphere microbiome. The illustration depicts the diverse community of microorganisms inhabiting the surfaces of plant leaves, distinguishing between beneficial microbes—symbiotic and commensal species (shown in blue)—and pathogenic microorganisms (shown in red). Both the adaxial (upper) and abaxial (lower) surfaces of leaves support distinct microbial assemblages, predominantly bacteria from the phyla Proteobacteria, Firmicutes, Actinobacteria, and Bacteroidota. The presence of pathogenic organisms, including bacteria, fungi, viruses, and protists, highlights potential threats to microbiome stability and plant health. A bar graph illustrates the relative abundance of dominant epiphytic bacterial groups, with Proteobacteria emerging as the most prevalent, followed by Firmicutes, Actinobacteria, and Bacteroidota. Understanding the ecological dynamics of these communities—including microbe-microbe interactions, niche specialization, and community resilience—is crucial for the rational design of synthetic microbial consortia (SynComs) aimed at enhancing plant protection, resilience, and overall health

The development of Phyllosphere-Modulating SynComs (PMS) is a promising strategy to harness the natural resilience of a microbial community for plant protection or improvement. Native phyllosphere bacteria have been shown to promote plant health by facilitating nutrient acquisition, producing plant hormones, and enhancing overall growth [18–25]. Although the phyllosphere is dynamic, microbial populations tend to stabilize over time, eventually forming a “core” microbiota [26, 27]. Non-pathogenic bacteria are the most abundant microorganisms associated with plant leaf phyllosphere, primarily dominated by Proteobacteria, followed by Actinobacteria, Bacteroidota, and Firmicutes [10, 28, 29] (Fig. 1). While fungi are generally less abundant on leaf surfaces, communities from the phyla Ascomycota and Basidiomycota also play important roles, particularly in nutrient cycling and pathogen suppression [28, 30, 31].

PMS can be developed using multiple strategies. The top-down approach involves starting with complex, naturally occurring microbial communities and simplifying these assemblages to study their structure, function, and ecological interactions. The bottom-up method focuses on selecting and combining specific beneficial microbes to achieve a specific outcome. Increasingly, integrated approaches that combine top-down, bottoms-up and emerging technologies, such as artificial intelligence, are being used to model microbial interactions, predict functional dynamics, and optimize SynCom stability and performance.

**Fig. 2:**
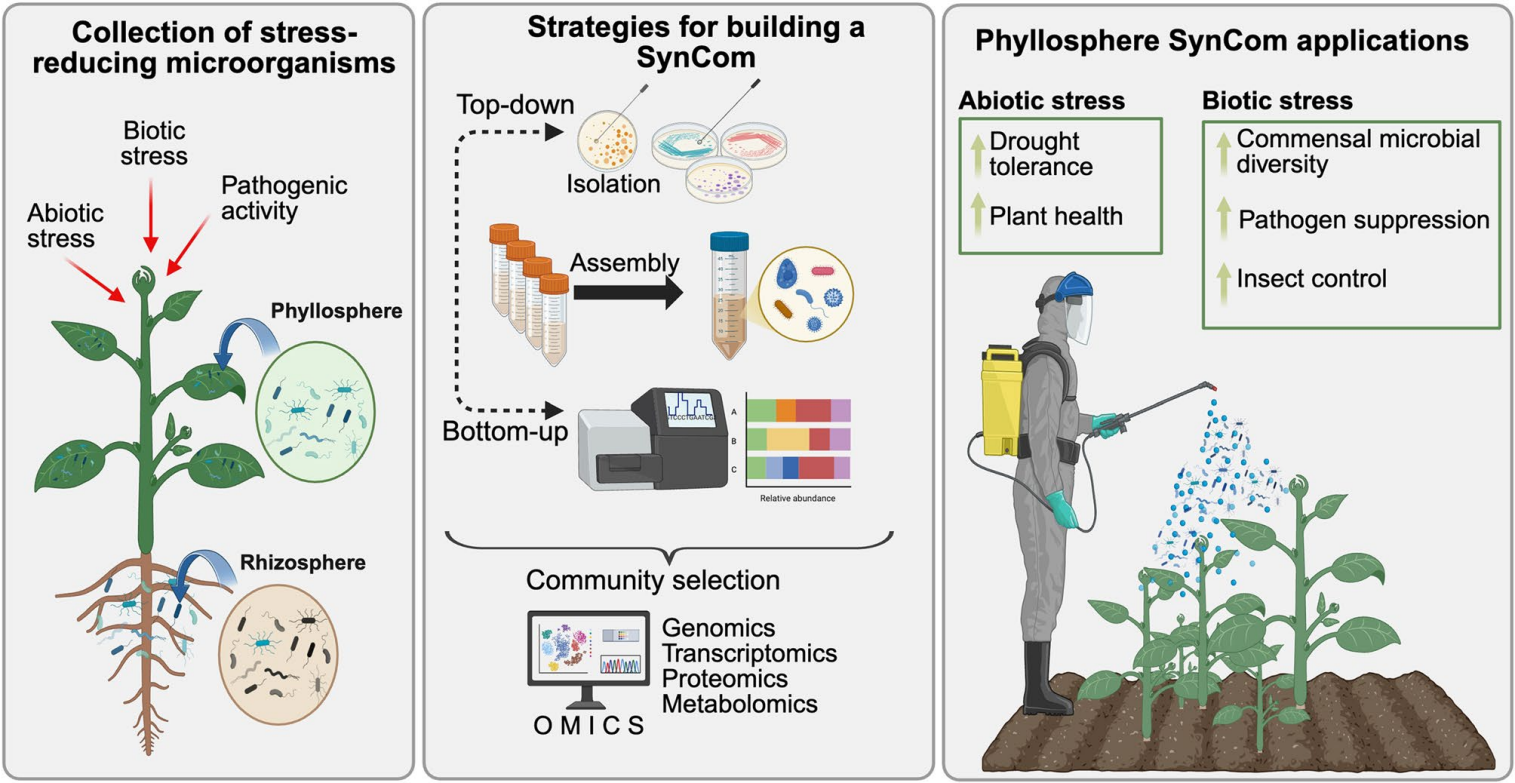
Graphical abstract highlighting the steps from isolate to application in PMS research. For PSM development, bacterial isolates are sourced from the host plant, a closely related wild relative, or from a biobank. To identify the most suitable isolates for the SynCom, both lab-based techniques and in silico bioinformatics approaches may be used. Once developed, the PMS is tested in greenhouse and field settings to assess its impact on plant health, as well as its ability to enhance abiotic and biotic tolerance and overall fitness. This figure outlines the process from isolate sourcing to SynCom testing and evaluation

This review consolidates recent literature on PMS developed using epiphytic bacteria, highlighting advancements in SynCom design, applications, and benefits to plant protection and stress resilience (Fig. 2). Despite notable progress, challenges remain regarding standardization and real-world implementation. We also discuss emerging research directions and advocate for open-access microbial databases and biobanks to facilitate the broader adoption of PMS in agriculture.

### Strategies for Building synthetic communities

**Fig. 3:**
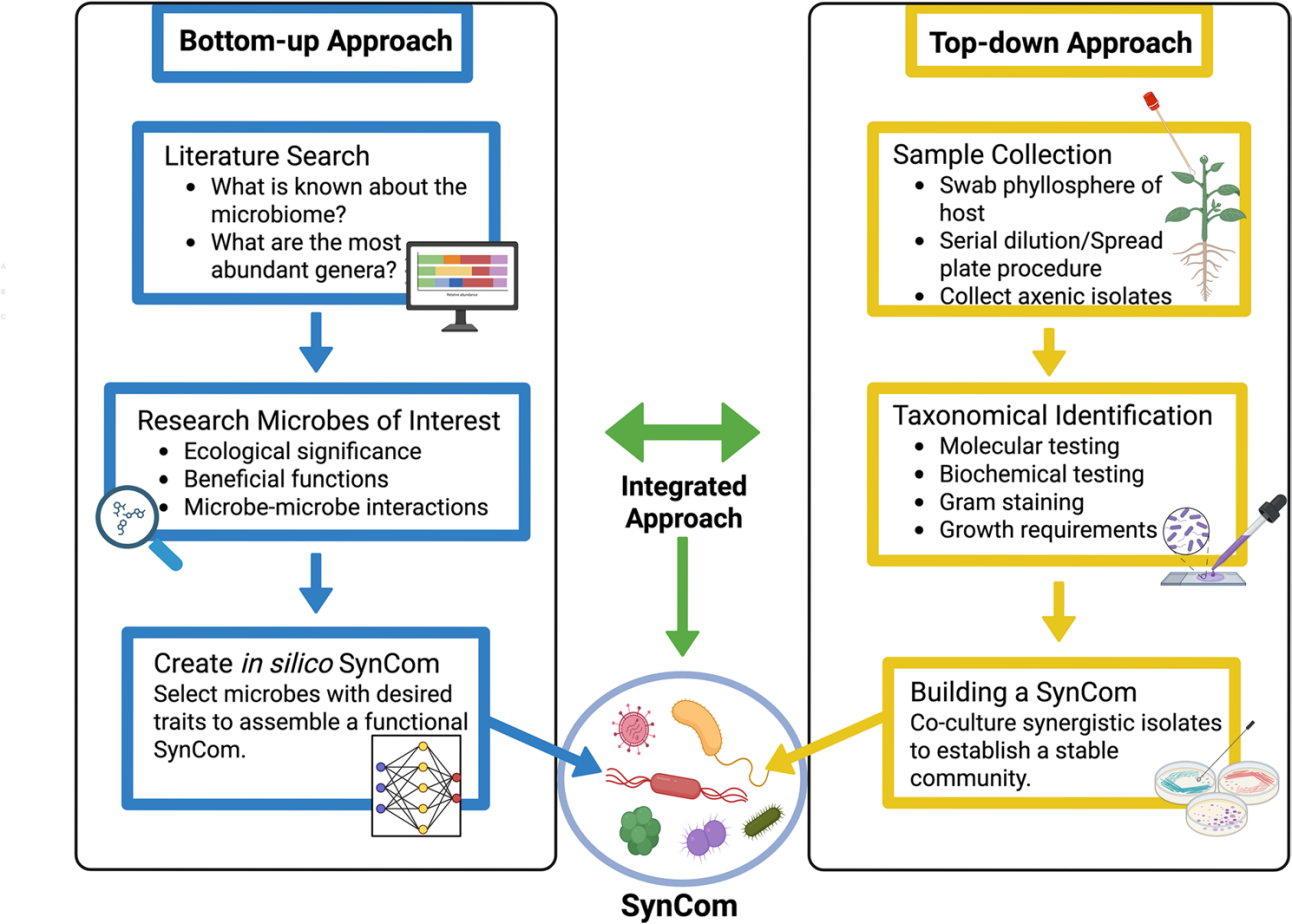
A schematic overview of the workflow for different SynCom developmental approaches. The top-down approach involves starting with complex, naturally occurring microbial communities and simplifying these assemblages to study their structure, function, and ecological interactions. The bottom-up method focuses on selecting and combining specific beneficial microbes to achieve a specific, targeted outcome. Integrated approaches combine top-down, bottoms-up and emerging technologies, such as artificial intelligence, to model microbial interactions, predict functional dynamics, and optimize SynCom stability and performance.

The process for isolate selection, the number of members included in a SynCom, and the strategy used to design a SynCom is unique to each specific study or application. When creating a PMS, researchers will often source bacterial isolates directly from the host plant, a closely related wild relative, or from a biobank (Table 1). SynComs are typically designed to achieve a specific goal, such as enhancing plant resilience to stress, boosting mineral accumulation, reducing insect pressures, suppressing pathogen abundance, or priming systemic resistance. The development of these SynComs generally follows either the bottom-up and top-down approach [32, 33], or, more recently, an integrated approach that combines elements of both strategies (Fig. 3). An integrated approach involves coupling microbiome sequencing data from the host environment (such as the plant leaf) with data from the isolated bacterial strains. This dual approach allows researchers to select species based on both their abundance and functional significance in the natural community.

Regardless of the approach used, during the assembly process, researchers must consider the functional roles of the strains. The interactions between strains should also be considered, as they should work synergistically to form a stable and functional community that mimics the ecological dynamics of the natural microbial community. Once assembled, the SynCom is inoculated into/on the plant, where it will be evaluated based on various performance metrics, such as plant growth, pathogen resistance, nutrient uptake, and other factors. In practical applications, PMS commonly include microbial members from the phyla Proteobacteria, Firmicutes, and Actinobacteria, which are favored for their ability to promote plant growth by secreting phytohormones, solubilizing nutrients, producing siderophores, and fostering beneficial microbe-microbe interactions [18–25]. Once constructed, SynComs are commonly suspended in an emulsified low nutrient mix or mineral carrier solution, dependent on the application situation [34]. Hand sprayers and backpack sprayers are the primary mode of application at rates of ~ 1 × 10^7^ CFU per milliliter [34]. Application efficacy of microbial suspensions may depend on the organism compatibility, climate variability, environment and microclimates, and specific host plant species [34, 35]. The performance of PMS should be monitored to determine short- and long-term success in terms of overall plant health but also the stability of the microbiota.

**Table 1:** Overview of approaches used for development of phyllosphere-modulating SynComs

**Approach used for SynCom**		**Method to obtain/identify bacterial isolates**	**# of isolates in SynCom**	**Reference**
**Bottom-up**		Literature Review of pathogen suppressing microbes	7	[25]
**Top-down**		Isolation	7	[23]
		16S rRNA sequencing		
		Isolation	48	[18]
		16S rRNA sequencing		
		Whole genome sequencing		
		Isolation	5	[21]
		16S rRNA sequencing		
		Morphological screening		
		Biochemical analysis		
		Isolation	4	[24]
		16S rRNA sequencing		
		Biochemical analysis		
**Integrated**		Previously characterized collection that was collected using the bottom-up approach	10	[22]
		Metagenomic analysis	12	[19]
		16S rRNA sequencing		
		Previously characterized collection	3	[20]

Table 1 summarizes published studies that have used Top-down, Bottom-up, or Integrated approaches to develop phyllosphere-modulating SynComs. Included are the number of bacterial isolates incorporated into each SynCom.

#### Top-down approach

The top-down approach begins with complex, native microbial communities. The goal is to simplify or manipulate the community while preserving its overall ecological structure and interactions. A sample is typically taken from the environment of interest such as swabbing the surface of a plant leaf to collect a representative microbial community [36]. Once the sample is collected, some microorganisms may be cultured using various media and growth conditions. Researchers may then use serial diluting and plating techniques to obtain pure axenic cultures, allowing individual isolates to be collected and studied independently. This process is important for characterizing cultivable members of the community, but a large portion of the microbiota remains unculturable, potentially leading to the exclusion of key species important for community function [37].

Once bacterial strains are successfully isolated, they undergo biochemical testing and molecular identification through methods such as 16 S rRNA sequencing. These approaches provide critical information about the bacterial species present and can reveal potential functional roles that each isolate might play within the community. The SynCom is then built by co-culturing synergistic isolates to establish a stable community with members sourced from the native microbiota.

#### Bottom-up approach

The bottom-up approach is a “function-first” strategy that enables researchers to investigate microbial interactions, ecological drivers, and functional roles within communities [35, 38–41]. The goal of this approach is to systematically design microbial communities by selecting and assembling individual strains with well-characterized, beneficial functions to achieve specific, targeted outcomes. This approach is particularly useful for studying processes like diversification, selection, and drift, which shape microbial communities [2].

The bottom-up approach is frequently used in *in silico* studies to analyze large-scale microbiome datasets and to model microbial community dynamics [9, 35]. These predictions can guide the rationale design of PMS, particularly when combined with experimental validation. Researchers use these techniques to classify microbial diversity, assess richness, and explore spatial and temporal dynamics within specific environments [2]. Based on these analyses, SynComs can be constructed using microbial taxa with key functional roles, selected for both ecological relevance and functional potential.

This strategy of functional tuning allows researchers to select key strains that provide targeted outcomes, such as plant growth promotion or pathogen protection [42, 43]. Microbes with low abundance are often considered inactive and may be excluded from the SynCom design [2]. A key advantage of the bottom-up approach is its ability to identify microbes with specific beneficial functions, such as biofilm formation, the production of secondary metabolites, or the emission of microbial volatile compounds that can enhance plant resistance [10, 39, 40, 44, 45].

#### Ecological and evolutionary foundations for phyllosphere SynCom design

A 2025 publication has highlighted the importance of grounding SynCom development in ecological and evolutionary theory to improve predictability and performance under field conditions [46–48]. The integration of these principles into SynCom design is increasingly recognized as key to enhancing community stability, functionality, and resilience, particularly in the phyllosphere [46, 47, 49]. In this environment, where colonization is constrained by abiotic stress and competition with resident microbiota, applying concepts from community assembly theory can improve SynCom establishment and performance [2, 46, 48]. Furthermore, mechanistic insights from frameworks such as priority effects, niche complementarity, and community coalescence provide guidance for understanding how microbial communities assemble and persist under natural conditions [2, 46, 50–52].

For example, priority effects suggest that early colonizing taxa can shape community dynamics, suggesting that the timing of SynCom introduction is critical for success [46, 51]. Niche-based theory supports the selection of functionally diverse strains with minimal resource overlap to promote stable coexistence and maximize trait expression on the plant surface [46, 53, 54]. The biodiversity and ecosystem function theory proposes that greater taxonomic and functional diversity enhances microbiome stability, resilience, and performance through niche partitioning and increased likelihood of including keystone taxa [46, 47, 50]. Including microorganisms with a known evolutionary history with the host plant, or sourced from the local environment, may also further improve SynCom compatibility, establishment, and persistence [55, 56]. By integrating the ecological and evolutionary theory into PMS design—alongside bottom-up, top-down, or combined approaches—researchers can develop more robust, ecologically relevant SynComs.

### Advantages of PMS for plant fitness and protection

SynComs applied to the plant phyllosphere offer a range of benefits by harnessing beneficial bacterial interactions that support plant health, resilience, and sustainability. The phyllosphere, which includes the aerial surfaces of the plant (like leaves and stems), is frequently exposed to environmental stressors and pathogens presenting a unique environment for SynComs to optimize plant functions. While some definitions of the phyllosphere include both epiphytic (surface-dwelling) and endophytic (internal) microbes, this work focuses on epiphytic communities. This distinction allows for targeted exploration of surface-associated microbial functions without confounding effects from endophytes. Recent studies highlight their promising applications in biocontrol, growth promotion, stress tolerance, environmental remediation, and as in situ positive controls for ecosystem research (Table 2).

**Table 2:** Applications of phyllosphere-modulating synthetic communities and effect on plant health

Application scenario	Plant species	SynCom source	SynCom composition	Applied concentration (CFU/mL)	Application effect	Reference
**Plant growth promotion**	*Agave tequilana*	In planta– Phyllosphere (Agave salmiana, wild relative)	*Belnapia rosea* MJ22 *Methylobacterium sp.* RAS18 *Micrococcus aloeverae* RAT4 *Nocardioides cavernae* AT2.17 *Staphylococcus hominis* AtDRG32 *Aureobasidium sp.* AT3.8 *Cladosporium sp.* cutV *Cellulomonas cellasea* RAS26 *Frigoribacterium endophyticum* AS3.20 *Sphingomonas aerolata* AS2.4 *Sphingomonas endophytica* AS3.13 *Sphingomonas melonis* AS2.3	10^6^	Improved plant productivity and overall plant health. Resembling wild type networks.	[19]
	Pak choy (*Brassica rapa* subsp. Chinensis)	In planta– Rhizosphere (*Cardamine violifolia*, a selenium (Se) hyperaccumulating plant)	*Bacillus subtilis* (Y1) *Bacillus cereus* (Y2) *Bacillus altitudinis* (Y5)	10⁷	Improved plant growth and nutritional quality by optimizing phyllosphere bacteria (specifically towards Se content)	[20]
**Abiotic stress**	Cotton (*Gossypium hirsutum*)	In planta– Phyllosphere from drought stressed plants	*Pseudomonas stutzeri* *Acinetobacter sp.* *Bacillus mojavensis* *Pseudomonas chlororaphis* *Enterobacter asburiae*	10⁹	Reduced drought stress under drought conditions.	[21]
**Biotic stress**	*Arabadopsis thaliana*	In planta–Phyllosphere (At-LSPHERE collection)	SynCom M10.35 (At-LSPHERE) *Pedobacter* Leaf194 *Chryseobacterium* Leaf404 *Curtobacterium* Leaf261 *Rhizobium* Leaf386 *Sphingomonas* Leaf29 *Plantibacter* Leaf171 *Brevibaccilus* Leaf182 *Sphingomonas* Leaf 37 *Methylobacterium* Leaf121 *Rhodococcus* Leaf 278	10⁷	Pathogen suppression against *Pseudomonas syringae* pv. tomato	[22]
	Willow (*Salix babylonica*)	In planta–Phyllosphere (Damaged leaves from beetle infested plants)	SynCom-mix7 *Pseudomonas psychrotolerans PSE37* *Pseudomonas psychrotolerans PSE38* 7 strains total used in consortia	10⁷	Biocontrol and reduced feeding of *Plagiodera versicolora* (Willow leaf beetle).	[23]
	*Arabadopsis thaliana*	In planta– phyllosphere (natural microbiome)	48 isolates (Firmicutes: ~20.8% Proteobacteria: ~62.5% Actinobacteria, Bacteroidetes, and other phyla)	10⁸	Reduced symptoms of necrosis and chlorosis caused by dysbiosis in the phyllosphere.	[18]
	Tomato *(Solanum lycopersicum*)	In planta Phyllosphere (Cytokinin enhanced plants)	*Pseudomonas aeruginosa* (IN68) *Ralstonia pickettii* (R3C) *Bacillus megaterium* (4 C) *Bacillus subtilis* (Bs SB491)	10^7^	Induced systemic resistance to foliar pathogens.	[24]
	Tomato *(Solanum lycopersicum* cv. Money maker)	Review of biocontrol studies - Koppert Biological Systems microbial collection	*Bacillus amyloliquefaciens* CECT 8238, *Bacillus amyloliquefaciens* CECT 8237, *Pseudomonas azotoformans* F30A, *Pseudomonas chlororaphis* MA 342, *Trichoderma harzianum* T22, *Trichoderma harzianum* ESALQ1306	10⁷	Pathogen suppression against *Botrytis cinerea* (up to 70%).	[25]

Summary of research publications related to phyllosphere-modulating synthetic communities, including the source of bacterial isolates, SynCom composition, application rates, host plants tested, and the effects of SynCom application on plant health and fitness

#### Enhanced plant growth

Numerous studies have investigated the plant growth promoting effects of rhizophore targeting SynComs in apple, corn, garlic, and wheat [10, 57–60]. These early works have paved the way for research investigating the use of PMS for plant promotion [35]. For example, a 2024 publication reported that *Bacillus subtilis*, *Bacillus cereus*, and *Bacillus altitudinis* enhance selenium (Se) absorption and macronutrient uptake in greenhouse grown pakchoi (*Brassica rapa* subsp. chinensis) [20]. In this study, plants treated with PMS had significantly higher biomass (40–70% fresh weight) and chlorophyll content (~ 15%), compared to control plants (no-microbial treatment) or plants treated with a single strain. SynCom inoculated plants also increased leaf Se content significantly (~ 50%) compared to the control plants. Upon further investigation, it was determined that the bacteria included in the SynCom not only secrete phytohormones and siderophores that enhance nutrient availability but modulated the microbial community, resulting in an increase in beneficial species of *Acinetobacter* and *Anaeromyxobacter*.

In another example, a field study on agave (*Agave tequilana*) demonstrated increased plant productivity following application of a SynCom composed of core phyllosphere microbiota from a wild relative, *Agave salmiana* [19]. Plants inoculated with the SynCom showed enhanced leaf development during the first year compared to uninoculated control plants. Moreover, sugar accumulation in the “piña” (stem) doubled from ~ 12 to ~ 21 °Brix on average two years post-inoculation, indicating the potential for long-term synergistic effects on plant growth and productivity [19].

#### Stress tolerance: abiotic and biotic

Abiotic stress, particularly drought, has a profound impact on agricultural productivity by disrupting plant growth, development, and yield. It is estimated that up to 70% of yield loss can be attributed to abiotic stress, with extreme weather conditions affecting approximately 90% of arable land worldwide [61, 62]. Phyllosphere microbiota have demonstrated significant potential in alleviating such stresses through diverse mechanisms, including the modulation of stress-related hormones, production of protective exopolysaccharides (EPS) and pigments, enhancement of nutrient availability, and activation of plant defense pathways [63, 64]. These mechanisms work in concert to improve plant resilience to environmental stress by directly influencing physiological and biochemical responses. While research focused on developing SynComs for plant protection is gaining traction, phyllosphere-specific applications are still relatively underexplored compared to the extensive research that has been performed exploring soil-based applications.

In a drought-sensitive variety of cotton (*Gossypium hirsutum*), a SynCom consisting of *Pseudomonas stutzeri*, *Acinetobacter sp.*, *Bacillus mojavensis*, *Pseudomonas chlororaphis*, and *Enterobacter asburiae* demonstrated significant drought stress mitigation compared to uninoculated cotton plants grown under the same drought conditions [21]. These bacteria, isolated from drought-prone regions, were selected for their osmotic stress tolerance and ability to produce compounds that reduce drought stress. Under drought conditions, the SynCom increased germination rates, root growth, proline accumulation, and overall biomass production. The consortium also modulated both ABA and ethylene pathways. This was achieved in part due to *Acinetobacter* producing ACC deaminase, which degrades 1-aminocyclopropane-1-carboxylate (ACC), reducing ethylene levels. Although ethylene is important for stress signaling at low concentrations, elevated levels can inhibit root and shoot growth. The SynCom also influenced cytokinin levels, promoting root elongation and improving recovery post-stress. The production of phytohormones, including auxins like indole-3-acetic acid (IAA), further promoted root elongation and improved water uptake. These combined mechanisms activated induced systemic tolerance (IST), priming the plant to better withstand abiotic stress. The diverse mechanisms by which SynComs mitigate stress warrant further exploration to better understand the pathways involved in plant–microbe interactions.

In addition to abiotic stresses, climate change has exacerbated biotic stressors, such as pests and diseases, which further threaten agricultural productivity and food security [62, 65]. Pathogen suppression is at the forefront for investigation as it is estimated that plant diseases account for billions of USD in economic losses across all crops [66]. The application of SynComs for pathogen suppression in soil is well documented, with the potential of PSM only just being realized [35, 58]. For example, Vogel et al. (2021) demonstrated the potential of phyllosphere-derived SynComs for foliar biocontrol in *Arabidopsis thaliana* using the At-LSPHERE collection [22]. This collection consists of 224 phyllosphere isolates, representing approximately 50% of the natural microbial diversity found in the plant’s microbiome [22, 67]. In their systematic analysis, protection against *Pseudomonas syringae* pv. *tomato* DC3000 varied with ~ 10% of the strains providing full protection, ~ 10% offering intermediate protection, and the remaining 80% showing little effect on disease progression. The most protective strains were distributed across various taxonomic groups, including Proteobacteria (e.g., *Pseudomonas*, *Rhizobium*, *Sphingomonas*, *Burkholderia*, *Erwinia*, *Serratia*, *Acinetobacter*) and Actinobacteria (e.g., *Arthrobacter*, *Curtobacterium*). Furthermore, experiments revealed both additive effects and the ability of single strains to confer full protection. Protection mechanisms varied, with some strains requiring pattern-triggered immunity coreceptor signaling, while others provided protection in the absence of functional plant immunity receptors BAK1 and BKK1. These findings suggest there is a functional advantage to selecting microorganisms that are adapted to the host plant, resulting in high microbial colonization densities (> 10⁵ c.f.u. mg⁻¹) [68, 69].

While the economic loss of crops due to insect damage is lower than that caused by other biotic stresses, SynComs may still be a valuable tool for enhancing plant resistance to insect herbivory [70, 71]. In field trials of *Salix babylonica* trees challenged by leaf beetles (*Plagiodera versicolora*), there was an enrichment of *Pseudomonas psychrotolerans* in both the phyllosphere and rhizosphere of beetle-damaged trees [23]. There were multiple strains of *P. psychrotolerans* found in association with the leaves which displayed insecticidal activity. Interestingly, *P. psychrotolerans* isolates from beetle-damaged (SynCom-SL) exhibited previously undocumented insecticidal properties. Based on these findings, the researchers created a curated SynCom, *SynCom-mix7*, composed of seven *P. psychrotolerans* isolates. When applied to willow leaves, this SynCom significantly reduced larval survival (~ 56%) compared to untreated controls. This systemic response to herbivory suggests that SynComs can be tailored to address both microbial and insect threats, thereby expanding their utility in integrated pest management strategies, both above and below ground [72, 73].

#### *Microbial symbiosis and functional diversity*

Microbial symbiosis plays a crucial role in plant-microbe interactions, with core bacterial communities establishing mutualistic relationships that benefit the host plant [26]. In contrast to single-strain inoculants, which lack the functional diversity needed to cope with stress, SynComs can offer redundancy and stability. This diversity not only boosts the plant’s adaptability to environmental challenges but also encourage the development of microbial communities that are more attuned to the plant’s native ecosystem, enhancing their long-term stability and effectiveness.

In a study of *Agave tequilana*, ten SynComs were developed and applied to six-month-old field-grown Agave plants. The plants were monitored over a two-year period. Interestingly, the SynComs designed around microbial hubs—groups of taxa associated with specific enriched microbial functions—resulted in microbial communities that showed greater alpha diversity, expanded metabolic functions, and more complex microbial networks [19]. After two years of growth, metabolic profiling revealed that plants treated with a SynCom were able to utilize a broader array of carbon sources, particularly carbohydrates and carboxylic acids, indicating enhanced metabolic flexibility. Further metabolomic analyses demonstrated that the SynComs modulated the plant’s metabolic profile, promoting the synthesis of bioactive compounds such as benzene derivatives and saponins—compounds with antifungal, antibacterial, and stress-resistant properties. These changes contributed to enhanced plant resilience. The findings from this study show that keystone taxa within a SynCom play a critical role in reinforcing beneficial microbial interactions and maintaining network complexity [51].

Recent research on the genetic determinants of microbiota composition further highlights the importance of maintaining balanced bacterial communities in plant leaves [18]. For instance, *Arabidopsis* mutants defective in pattern-triggered immunity and the MIN7 vesicle-trafficking pathway display leaf-tissue damage associated with dysbiosis, reducing plant fitness. This dysbiosis was marked by a significant shift in microbial composition, with a reduction of beneficial Firmicutes and Actinobacteria and an increase in Proteobacteria. To restore microbial balance, a synthetic community was developed from bacterial isolates, including Proteobacteria, Firmicutes, and Actinobacteria, from the phyllosphere of healthy wild-type *Arabidopsis* (*Col-0*) plants. The application of the PMS led to restoration of microbial diversity, demonstrating the potential of SynCom-based strategies to mitigate microbial imbalances and promote plant health [18, 74].

## Competitive interactions and pathogen suppression

The phyllosphere is a resource-limited environment, densely populated by microbial communities, where bacterial competition drives either exclusion or co-existence based on fitness differences and resource overlap [69]. In this context, tailor-made PMS engineered to promote specific microbial groups or functions can shift the microbial dynamics in favor of beneficial microorganisms. For example, the application of SynComs can help suppress pathogen establishment by allowing beneficial microbes to outcompete harmful microbes for space and nutrients on the leaf surface [44, 51, 75]. Additionally, SynComs can be engineered to boost antibiosis, leveraging the combined antimicrobial activities of multiple beneficial microbes—such as the production of bacteriocins or antibiotics—to more effectively inhibit pathogen growth [56, 76].

In one study, *Arabidopsis thaliana* [22] plants were inoculated with individual strains of bacteria and subsequently challenged with a luxCDABE-tagged pathogen after colonization. This approach allowed for precise quantification of pathogen colonization via luminescence and disease symptom scoring. Remarkably, about 8% of the screened isolates conferred full protection, as evidenced by disease phenotypes that closely resemble those of uninfected plants and significant reductions in pathogen luminescence. The most effective protective strains belonged to the genera *Pseudomonas* and *Rhizobium*, which achieved high colonization densities critical for competitive exclusion of pathogens. Furthermore, when these protective isolates were assembled into a SynCom colonization densities frequently exceeded that achieved by individual strains.

In another study, researchers evaluated whether directly spraying selected pathogen-suppressing bacteria onto tomato leaves could effectively protect plants against *Botrytis cinerea* through direct antagonism via antimicrobial peptides [25]. The SynCom developed included strains from the Koppert Biological Systems collection with complementary functions and was applied at a concentration of 1 × 10⁷ CFU per plant (Table 2). While the individual strains *Pseudomonas chlororaphis* and *Pseudomonas azotoformans* successfully reduced necrotic lesion areas by up to 56%, while those treated with the SynCom ranged from 50 to 70%. The most successful SynCom, SynCom2B was composed of six antimicrobial (surfactin, fengycin, iturin, phenazines, pyrrolnitrin, pyoluteorin, and peptaibols) producing bacteria: *Bacillus amyloliquefaciens* CECT 8238 and CECT 8237, *Pseudomonas chlororaphis* MA 342, *Pseudomonas azotoformans* F30A, and *Trichoderma harzianum* T22 and ESALQ1306 [77–81].

The use of biofilm-forming SynCom members on leaf surfaces can also serve as a physical barrier to inhibit pathogen invasion or prevent pathogens from spreading [10, 82]. These biofilms can also help in the retention of beneficial plant-associated microbes, ensuring a prolonged protective effect [82, 83]. These results validate that inoculation with a diverse PMS can create an environment of competitive exclusion in the phyllosphere.

### Induced resistance and immune system priming with SynComs

The application of beneficial microorganisms has been shown to trigger the plant’s immune system through induced resistance [10, 84–86]. This process activates defense pathways that enhance resistance to pathogens, such as by initiating induced systemic resistance (ISR). When ISR is activated, plants send long-distance signals that prompt immune responses in distant tissues, providing protection against pathogen invasion [85]. SynComs are also effective at inducing a priming effect, where the diverse microbial community prepares the plant’s immune system to respond more rapidly and effectively to future pathogen attacks [87]. By introducing a variety of beneficial microbes that interact synergistically, the application of PMS can help the plant recognize potential threats and respond faster without requiring a full immune activation [87]. This microbial memory improves the plant’s resilience over time, providing long-lasting protection against pathogens and environmental changes with a more efficient immune response.

Gupta et al. (2021) demonstrated that applying a SynCom composed of *Bacillus megaterium*, *Bacillus subtilis*, *Ralstonia pickettii*, and *Pseudomonas aeruginosa* to the tomato (*Solanum lycopersicum*) phyllosphere can promote induced systemic resistance (ISR) against foliar pathogens *B. cinerea*,* X. euvesicatoria* and *O. neolycopersici* [24]. Tomato plants with elevated cytokinin (CK) levels were used as a model to recruit beneficial microbiota, with dominant Bacilli members subsequently incorporated into the SynCom. To understand the underlying mechanism, plants pre-treated with these bacteria were subsequently challenged with wounding or with immunity elicitors. Plants treated with *Bacillus megaterium* 4 C and *Bacillus subtilis* Bs SB491, showed significantly increased ethylene production and activation of key defense genes (salicylic and jasmonic acid) compared to plants treatments with only *Bacillus megaterium* 4 C. In contrast, Gram-negative isolates such as *Ralstonia* and *Pseudomonas* elicited a weaker immune response and did not induce activation of plant defense mechanisms. ISR was observed under both soil and sterile conditions, though the response was weaker in sterile conditions. Furthermore, on-plate biocontrol assays confirmed that none of the beneficial bacteria directly inhibited pathogen growth, supporting the conclusion that ISR via defense priming, rather than direct antagonism, was the primary mode of action. These findings highlight the role of CK as a key regulator of phyllosphere microbial composition, selectively enriching immunity-inducing Bacilli through structural and chemical pathways [24]. While other studies highlight key interactions between microbiota and host phytohormones, Gupta et al. (2021) provides a specific example of SynCom development targeted for induced systemic resistance (ISR) in cytokinin-enriched tomato plants [11, 88].

#### Metabolic flexibility and resource use efficiency

PMS can be designed for metabolic flexibility and resource use efficiency, which is advantageous for improving plant fitness in nutrient limited or environmentally challenging environments. The phyllosphere, with its limited and highly heterogeneous resources, drives metabolic overlap among microbes, fostering competition and niche occupation [89]. This ecological dynamic suppresses pathogens by limiting available resources, making phyllosphere-targeted SynComs effective at outcompeting harmful microorganisms. Beneficial microbes that share ecological niches with phytopathogens can act as natural biocontrol agents, effectively reducing pathogen load and promoting plant health [69, 89, 90]. The metabolic flexibility of these communities is particularly important in promoting nutrient acquisition and enhancing plant resilience to environmental stresses, especially in organic and low-input farming systems where chemical inputs are minimized.

Although studies are lacking specific to the phyllosphere, evidence from rhizosphere-SynCom experiments suggest the microbial consortiums can improve resource use efficiency by optimizing microbial interactions and nutrient cycling [35, 91]. For example, a study on cotton revealed that a rhizosphere-targeting SynCom significantly improved metabolic flexibility by increasing soil nitrate availability (by 28% at the early growth stage and 55% at flowering) and enriched nutrient-solubilizing bacterial taxa (Actinobacteria, Firmicutes, and Cyanobacteria). These microbial shifts fostered beneficial interactions, leading to improved plant fitness and yield [91]. A similar approach in the phyllosphere, where microbial communities are designed based on plant exudates (carbohydrates, amino acids, and volatile organic compounds), could optimize resource use efficiency, microbial colonization, and competition [54, 90, 92, 93]. By harnessing these microbial interactions, PMS could enhance resource efficiency, echoing the benefits observed in the rhizosphere.

#### In situ control for research

The phyllosphere has emerged as a valuable model system for studying plant-microbe interactions and host-microbe relationships [29]. Phyllosphere-modulating SynComs are increasingly used as in situ positive controls in studies investigating plant-microbe dynamics within natural ecosystems. By introducing a SynCom with known microbial diversity and functions into field environments, researchers can monitor and quantify specific microbial roles and interactions under real-world conditions [94]. This approach provides deeper insights into how individual bacterium and microbial consortiums contribute to plant health, such as enhancing nutrient uptake and immune responses [94, 95].

PMS may also be used as a control in a scientific experiment by serving as a standardized, known microbial consortium with well-characterized functions, allowing researchers to compare the effects of manipulating or introducing new variables, such as specific microbes or environmental conditions, into the system [96, 97]. For example, a PMS with a defined, stable composition can also be used as a baseline to measure the impact of specific changes in the experimental setup. These controlled studies provide valuable insights for predictive ecology by demonstrating how specific microbial communities can enhance plant performance and stress tolerance under challenging conditions [97–99].

#### Nutritional and health promoting benefits

Emerging research on plant-microbiome interactions has shed light on the role of microbial communities in modulating nutrient bioavailability, which directly impacts both plant and human health [5, 100]. The rhizosphere and phyllosphere harbor diverse microbial taxa that influence nutrient uptake, metabolism, and assimilation through biochemical transformations such as nitrogen fixation, phosphate solubilization, and micronutrient mobilization [10]. By selectively enriching beneficial microbial taxa, PMS can be engineered to enhance specific nutritional traits in crops offering a targeted approach to improving the nutritional quality of food [20, 43, 100].

He et al. (2024) provide empirical evidence supporting the feasibility of SynCom-based nutritional enhancement strategies, further underscoring their potential role in biofortification efforts [20]. Their study demonstrated that a plant-specific SynCom (PSM) significantly increased Se content, vitamin C concentration, and soluble protein levels in pakchoi (*Brassica rapa* subsp. chinensis), illustrating the potential of microbiome-mediated interventions to fortify essential nutrients in edible crops [20]. Compared to traditional Se fortification methods such as soil amendments and foliar sprays, which can be costly, inefficient, or environmentally degrading, the application of a PSM presents a more sustainable and effective alternative [101]. While the initial results provide a promising framework for leveraging SynComs to enhance the nutritional content of food crops, additional studies are necessary for optimization and validation [100].

### Limitations and challenges of SynCom application

#### Isolation and identification of bacteria

One of the biggest limitations of using SynComs for plant protection is the issue of bacterial culturability. This issue not only hinders SynCom development but also poses a broader challenge for microbiome research [41–43]. It is estimated that less than 2% of microorganisms can be cultured in laboratory conditions, which limits both the complexity of SynComs that can be developed and their ecological relevance to the host plant [102]. Furthermore, while the cost of sequencing has drastically decreased, accurately assessing microbial diversity within a SynCom remains difficult due to the limitations of low-resolution, short-read sequencing techniques commonly used in research studies. These methods often fail to identify microbes at the species level, reducing the resolution at which microbial communities can be characterized. The challenge is further exacerbated by incomplete microbial databases, which lack comprehensive data for many bacterial species. As a result, taxonomic identification may be imprecise or unattainable, leading to the underrepresentation or misidentification of microorganisms. To address this, more support is needed for the sequencing and cataloguing of isolates and subsequent submission of these findings to publicly available databases. These efforts will not only expand our knowledge of the microbial world but will improve the accuracy of taxonomic assignments and provide a more reliable framework for SynCom and microbiome research.

#### Stability of SynComs

Another limitation of using SynComs in agricultural applications is the instability of microbial communities once applied to plants. Achieving a consistent microbial community, particularly in field settings, is difficult due to various factors, such as fluctuating environmental conditions and plant health, which can alter plant-microbe interactions [44–46, 103]. For instance, abiotic factors such as temperature and soil moisture, as well as biotic factors like the plant’s growth stage or pathogen pressure, can influence the composition and function of microbial communities. Although there are studies exploring how these factors impact microbial communities and technological strategies for enhancing stabilization [103], there remains a need for more robust methods to maintain SynCom consistency over time. Efforts to resolve this issue include the development of microbial strains with enhanced resilience to environmental stressors, such as the use of engineered bacteria that produce protective metabolites (e.g., exopolysaccharides) that enable environmental adaptation, even in drought or extreme temperatures [88].

Additionally, biodegradable encapsulation technologies are being explored to protect microbial communities during application, ensuring that SynComs remain stable and active under harsh field conditions [104–106]. Natural polymers such as alginate, chitosan, starch-based biopolymers, and their composites (e.g. alginate–bentonite or alginate–pea protein blends) are widely used to embed beneficial bacteria in protective microcapsules [104–108]. These capsules slow moisture loss and buffer temperature changes, preserving cell viability, which has shown promising results for improving SynCom performance in field setting. In addition, researchers have demonstrated calcium-alginate hydrogel beads can act as a UV shield [106, 108]. Encapsulating *Bacillus-* based biopesticides in alginate significantly reduces UV degradation and heat inactivation, yielding higher post-exposure bioactivity than freely suspended cells [108]. Additionally, functional persistence can be improved by gradually releasing bacterial cells, which helps maintain their presence on the phyllosphere for extended periods [108].

A major challenge in applying encapsulated SynComs to the phyllosphere is ensuring they firmly adhere to leaf surfaces and resist removal by rain or irrigation. Tailor-made capsule designs have been developed to address this challenge, such as coating alginate beads with chitosan, a cationic, film-forming biopolymer [107]. This approach improves SynCom adhesion to the waxy cuticle and enhances capsule stability against environmental stressors such as moisture, temperature, and wind [106, 107]. It has also been shown that encapsulating bacteria such as *Pseudomonas putida* in an alginate–bentonite composite, results in higher survival rates and more effective phyllosphere colonization [105, 108].

Bioinspired inspired modifications to encapsulation systems could further enhance foliar retention and colonization efficacy. For example, spiny microcapsules with rough, pollen-like surfaces have been shown to mechanically interlock with a leaf surface, reducing wash-off by 12.5-fold compared to smooth encapsulations [109]. Although this method was originally developed for foliar micronutrient applications (e.g., Fe), it offers valuable insight for advancing microbial delivery technologies. Nevertheless, optimizing encapsulation composition and delivery conditions remains essential for each application. Spray parameters (droplet size, timing) and capsule characteristics (size, degradability, nutrient additives) must be evaluated under realistic field conditions to maximize retention and microbial efficacy [104, 105, 110]. Continued efforts are needed to enhance biodegradable encapsulation systems, focusing on formulation refinement and controlled release kinetics [104–106]. 

#### Microbial evolution and long-term effectiveness

The rapid evolution of bacteria presents a significant challenge to the stability and long-term effectiveness of PMS. Microbes can undergo rapid mutations, which can lead to genetic divergence from the originally intended strains within a SynCom. This evolution may alter their behavior, interactions, or ecological roles, undermining the SynCom’s reliability and effectiveness over time [111]. The potential for such evolutionary shifts introduces a risk that SynComs may not maintain their intended performance, particularly in dynamic, real-world environments.

To mitigate this issue, several strategies have been proposed. One promising approach involves engineering “evolution-proof” microbes through synthetic biology techniques. By designing genetic circuits or regulatory networks that allow microbes to sense and respond to environmental changes, these engineered strains can adapt to evolving conditions without losing their intended functionality or ecological role [112]. Such circuits could activate specific stress-response pathways or enhance resilience to environmental shifts, maintaining microbial performance in the face of selective pressures. Another strategy focuses on the development of PMS with a broad functional diversity. By ensuring that the microbial community has a diverse range of functional capabilities, the overall stability of the SynCom could be better preserved, even if individual strains mutate or evolve. This redundancy in function means that if one strain’s performance is compromised due to genetic changes, other microbes in the community can compensate, preserving the SynCom’s overall efficacy.

### Emerging tools to improve PMS

One emerging tool for SynCom develop is Clustered Regularly Interspaced Short Palindromic Repeats (CRISPR), a powerful genetic engineering tool widely employed in synthetic biology. CRISPR enables precise modifications to an organism’s genome, allowing for targeted alterations in gene expression. For example, microorganisms can be engineered to produce specific secondary metabolites (SM) that boost their capacity to combat targeted plant pathogens and pests, or to optimize their interactions with host plants [8, 113, 114]. Although genetically engineered (GE) studies have predominantly focused on well-characterized biological control isolates such as *Beauveria bassiana* and *Trichoderma reesei*, there is potential to integrate SynCom-based approaches to optimize plant-microbe interactions and functional benefits for desired secondary metabolite production [114, 115]. Additionally, CRISPR could enable the incorporation of quorum-sensing circuits in SynComs, facilitating communication between bacterial strains to synchronize their activities. This would allow for the activation of beneficial responses, such as pathogen suppression or nutrient release, precisely when needed, conserving both plant and microbial energy [35, 56, 116]. While GE raises environmental concerns in microbial engineering, research has focused on developing effective “kill switches” to mitigate the risk of environmental contamination from GE microbes, demonstrating biocontainment strategies in the relevant genus *Pseudomonas* [114, 117, 118].

Another key advancement involves the use of artificial intelligence (AI) and machine learning (ML) for advancing SynComs for plant protection. Although AI and ML are still emerging in plant science, their potential to revolutionize microbiome research is evident from their success in other life sciences [119]. AI and ML can assist in optimizing the design of SynComs by analyzing large volumes of data to predict the most effective microbial community compositions for specific plant protection goals [6, 119]. Building on these advancements in microbial engineering and biocontainment, emerging computational technologies are now being explored to further optimize SynComs for improving plant health and productivity.

This computational analysis will be complemented by in situ methodologies, bridging the gap between theoretical design and practical application. The combination of these advanced approaches will enable deeper insights into SynCom functionality, ultimately accelerating the development and deployment of SynComs for sustainable agricultural practices.

### Future perspectives

#### Microbial community standards and shared resources

Establishing standardized microbial communities is essential for ensuring reproducibility, comparability, and scalability in SynCom research and application. The lack of a common framework makes it challenging to evaluate the true potential of SynComs for plant protection [95]. Collaborative efforts, such as open-access biobanks and databases, could enable the widespread sharing of phyllosphere isolates, associated microbial data, and experimental protocols. These efforts would facilitate validation across various plant species and agricultural contexts.

#### Spatial characterization

One hurdle for advancing SynCom research lies in the spatial complexity of microbial interactions, both within the plant and in the surrounding environment. To develop successful SynComs for plant protection, it is critical researchers elucidate how these communities interact with plants at different growth stages, across various tissues and in response to different environmental conditions (e.g., temperature, humidity, soil pH). This spatial complexity involves both intra- and inter-species interactions and understanding these dynamics could open new possibilities for fine-tuning microbial formulations that work optimally under specific environmental conditions.

#### Holistic studies

While much of the current research on PMS focuses on short-term plant health outcomes, the long-term effects of these synthetic communities must also be considered [19]. Future research projects should investigate interactions between microbial communities and plant health over extended periods, as well as their broader ecological impacts. This is important as plants are not grown in isolation but are part of a complex, integrated ecosystem that is constantly changing. As such, the influence of PMS on the surrounding ecosystem, such as the soil microbiome and nutrient cycling should be taken into consideration. Results from these studies will better inform the impact of SynComs more broadly on agricultural systems.

### Expanding research of non-model organisms

Of the eight PMS scientific articles reviewed (Table 2), 25% of the studies were conducted using the model-organism *Arabidopsis thaliana.* Although informative, the use of model organisms (grown in controlled environments), does not always reflect the complexities of real-world crops. Additional studies are needed using ecologically relevant field-grown crop species to accurate assess the short- and long-term effects and stability of PMS applications. By expanding research to diverse plant species, scientists can better evaluate the efficacy and safety of SynComs under a wider range of conditions, ensuring that the findings are applicable to a broader spectrum of crops.

## Conclusions

PMS developed using epiphytic bacteria offer a promising strategy for enhancing plant health, stress resilience, and crop yield. Current literature shows that PMS typically consist of bacterial phyla such as Proteobacteria, Firmicutes, and Actinobacteria. However, the absence of standardized protocols for PMS development, application, and performance assessment continues to hinder meaningful comparisons across studies and limits broader adoption.

To advance the development and effectiveness of PMS, future research should integrate emerging technologies—such as machine learning, high-throughput sequencing, and metabolomics—into SynCom design. These tools can reveal complex microbial interactions and enhance community stability. Moreover, experimental approaches should reflect real-world agricultural conditions, including crop-specific environments, seasonal variability, and long-term field trials.

Equally important are collaborative efforts to establish open-access databases and biobanks of well-characterized, plant-associated microbial strains. These shared resources will be essential for improving the reproducibility of SynComs and accelerating the translation of PMS research into practical agricultural solutions.

## Data Availability

No datasets were generated or analysed during the current study.

## Abbreviations

AI: Artificial intelligence
ACC: 1-Aminocyclopropane-1-carboxylic acid
CFU: Colony forming unit
CK: Cytokinin
CRISPR: Clustered regularly interspaced short palindromic repeats
EPS: Extracellular polymeric substances
GE: Genetically engineered
IAA: Indole-3-acetic acid
ISR: Induced systemic resistance
IST: Induced systemic tolerance
ML: Machine learning
PMS: Phyllosphere-modulating SynComs
SM: Secondary metabolites
SynCom: Synthetic microbial community
